# Addressing Stigma Relating to Mental Illness in Low- and Middle-Income Countries

**DOI:** 10.3389/fpsyt.2015.00038

**Published:** 2015-03-11

**Authors:** Franco Mascayano, Julio Eduardo Armijo, Lawrence Hsin Yang

**Affiliations:** ^1^School of Public Health, Faculty of Medicine, University of Chile, Santiago de Chile, Chile; ^2^Faculty of Medicine, University of Santiago, Santiago de Chile, Chile; ^3^Department of Epidemiology, Mailman School of Public Health, Columbia University, New York, NY, USA

**Keywords:** stigma, mental illness, developing countries, interventions, gap treatment

## Introduction

Mental health disorders (especially psychotic disorders such as schizophrenia) constitute the leading cause of disability-adjusted life years (DALYs) from all non-communicable diseases (1). These disorders relate to severe social, educational and occupational impairment, physical illness, and premature mortality (2). Approximately, three-quarters of the burden of mental illness comes from low- and middle-income countries (LMICs) (3).

Governments from LMICs spend the lowest percentages on mental health worldwide (4). Most of these countries model their mental health systems based on care that is primarily delivered through psychiatric institutions. Hence, the implementation of community mental health models has been extremely difficult, given that attaining the additional funds required to establish outpatient services has frequently not been available in these countries. Additionally, human resources are limited and inequitably distributed. This explains why in LMICs more than 75% of people that require mental health care do not receive any kind of intervention (5). This statistic refers to the “treatment gap”: the proportion of people who need but do not receive care. The World Health Organization (WHO) has reported that the treatment gap for serious mental disorders is 35–50% in developed countries and 76–90% in LMICs (6).

Stigma comprises a major problem related to help seeking in people with mental health difficulties in developing countries. Stigmatized individuals may anticipate devaluation and discrimination from others, leading them to adopt harmful coping mechanisms such as secrecy or withdrawal (7). Studies conducted in LMIC such as Ethiopia and India have reported this tendency, whereby consumers prefer to not disclose their problems or symptoms with health professionals or even with their own relatives (8). Such harmful coping strategies further obstruct effective treatment use. Several authors have recommended the implementation of national public education campaigns and local interventions to increase mental health literacy to reduce discrimination in these countries (9).

## Mental Illness Stigma in Developing Countries

The lack of investment in mental health services in LMICs has been attributed not only to scarcity of funding but also to the absence of interest about those with mental illnesses. Therefore, understanding stigma – both at the community and the institutional decision-making level – is a fundamental step to improving mental health services and policies in these countries (10). A qualitative study including 50 academics and policy makers from developing nations concluded that stigma occurs at a comparable level within the general population, health sectors, and policy makers (11).

While stigma is a universal phenomenon, stigma appears to be a stronger barrier to treatment access within low-resource areas and among vulnerable members of the population including the poor, women, and ethnic minorities (12). For instance, in India, people with schizophrenia have reported high rates of perceived stigma and they have primarily felt that discriminatory attitudes come from community (46%) and from family members (42%) (13). Related to help seeking, Uribe and colleagues conducted a qualitative study with 52 consumers and 18 relatives in Colombia. Participants described their stigma experiences as taking place via mechanisms of rejection, ignorance, and derogatory language, which led to them not disclosing their psychiatric status with anyone (14). In Nigeria, 103 people with major depressive disorders were interviewed. Concealment of mental illness was most common due to anticipated discrimination (51.5%) in this population. Younger people (age <40 years) with a higher level of education appeared to be at high risk for experienced discrimination as well (15). Finally, in Ethiopia, 75% of relatives of consumers with psychiatric diagnosis (psychotic and mood disorders) reported they had experienced stigma via association with their ill relatives and 37% wanted to conceal the fact that a relative was ill (8). While there are important cultural variations across contexts, these studies’ findings roughly cohere with the main conclusions of one of the largest mixed-methods studies of stigma and discrimination faced by individuals with schizophrenia (*n* = 282) and caregivers (*n* = 282) in a LMIC setting (India) (16). This study found that while experiences of negative discrimination were not infrequent (42%), instead it was internalized forms of stigma (e.g., a sense of alienation) that predominated (79%) and were powerfully associated with impaired role function.

## Addressing Stigma and Discrimination

Considering the influence of stigma in health seeking, many LMICs have declared that the implementation of anti-stigma interventions must to be a priority for health policy (17). Some national programs have been implemented, focusing principally on improving knowledge about mental health as well as attitudes toward individuals with psychiatric disabilities.

For instance, Petersen at al. evaluated the integration of a mental health team into primary care in South Africa and Uganda. The authors interviewed several stakeholders (professionals, community mental workers, and consumers) and identified positive effects in healthcare professionals’ attitudes as well as in connecting consumers with health services and self-help groups (18). In the same line, the intervention used by Makanjuola and colleagues was developed to train primary care workers in LMIC’s as a result of having few local human resources. In this study, 24 (mostly female) community health workers participated, and after intervention, showed improved attitudes toward people with mental illness (19). Similar programs were conducted in Iraq and India, where programs aimed toward healthcare professionals and non-professionals have demonstrated utility in connecting health workers and consumers to each other (20).

In comparison with national and regional campaigns conducted in high-income countries, the development of anti-stigma programs is still scarce in LMICs settings. Programs such as “Like Minds Like Mine” (New Zealand) or “Opening Minds” (Canada) are examples of best practices in this field, contributing to increase understanding, tolerance, and care-taking of people with mental illness. Despite these noteworthy goals, one of the biggest unanswered questions in regard to these campaigns has been the equivocal reduction in stigma outcomes (21). Some authors have suggested possible explanations, including a lack of consistent evidence to support the premise that these massive campaigns (e.g., social marketing) comprise the appropriate approaches to eradicate stigma (22). Finally, the cost of these campaigns might be unrealistic for settings with fewer resources. Therefore, taking advantage of natural facilitators and cultural dynamics that might identify particular domains of stigma (e.g., “What Matter Most”) within each community among LMICs could be extremely helpful for implementing these kinds of interventions (23, 24). For example, “what matters most” within India has been identified as meeting role expectations specific to gender in regards to work and marriage (whose economic importance is magnified in the context of frequent poverty), and adhering to codes of conduct and socially acceptable behavior as dictated by traditions of Dharma (16). Messages to reduce public stigma in the Indian context might therefore be most effective if they promoted the possibility that individuals with schizophrenia can attain these role expectations signifying “what matters most” with proper supports (25, 26), rather than messages aimed at generic stereotypes (e.g., “people with mental illness are not dangerous”).

## Lessons from Developing Countries

There are many barriers in LMICs in the implementation of successful anti-stigma programs: these include low rates of psychiatric help seeking (that may be influenced by common cultural beliefs about traditional medicine), domestic violence, gender dynamics (i.e., “Machismo” or “Culture of Honor” within certain cultural groups) (27), lack of political prioritization of mental health care, low financial investment on health, and material poverty and social marginalization (10).

In response, Syed at al. have highlighted that there are key health areas where developed countries can learn from experiences in the developing world (28). This includes domains of mental health delivery that shape the formation of stigma, such as rural health service delivery; successful task-shifting services; delivery of counseling and case management by peers and health workers (29); innovative and low cost use of mobile phones; local product manufacture and economic development (e.g., tourism industry in Kenya) (30); and social and community entrepreneurship (31), among others.

Additionally, in developing countries, there is a particular concern in reducing cultural, social, financial, or gender-related barriers to service delivery. This has been reflected in the implementation of valuable family and community-based interventions to manage serious mental disorders, using de-stigmatizing strategies such as therapeutic optimism, the extension of support networks, and the connection with formal and informal social services (32). The “what matters most” concept (23) may be particularly useful here, in helping consumers to achieve the capacities essential to being viewed as a “full person” within their contexts. Finally, considering biosocial complexity of mental disorders in specific cultures can be essential to generate appropriate approaches to make diagnostic assessment and therapeutic innovation in those countries (10).

## Conclusion

We point out that stigma remains an unresolved problem in LMICs. Despite the few initiatives that have been promoted, the establishment of rigorous evaluation is needed. Although there are valuable experiences to be learned from national campaigns in high-income countries, extrapolation of those programs to LMIC’s is not feasible considering the required resources. For that reason, we strongly encourage leveraging natural facilitators, cultural influences, and potential resources in developing nations. Also, there are outstanding experiences delivering health education to reduce stigma for communicable and non-communicable diseases in poor resource contexts (e.g., HIV) (33), which might provide powerful insights to future approaches.

In sum, there are specific factors within developing countries that, despite showing substantial stigma and discrimination as described above, may contribute to favorable implementation of anti-stigma projects (32). Some examples include (a) communities that are more able to tolerate and protect consumers (e.g., within the Jamaican population) (34); (b) social solidarity by offering work opportunities in local businesses (e.g., tribal or village associations in Latin America) (35); (c) participation in traditional and religious healing rituals such as musical rituals in Sudanese culture (36); (d) more flexible job requirements (e.g., agrarian work within rural China) (37); (e) family and extended kinship or a communal network to support individuals with a mental disorder (e.g., kinship ties in Ethiopia and Tanzania) (38); and (f) attribution of cultural or spiritual value to psychotic experiences such as visions, or what might be interpreted as prophetic encounters (e.g., Uganda) (39). We propose that to most effectively implement anti-stigma interventions in LMICs, interventions might best leverage these existing strengths to combat the severe mental illness stigma and discrimination that occurs within these contexts.

## Conflict of Interest Statement

The authors declare that the research was conducted in the absence of any commercial or financial relationships that could be construed as a potential conflict of interest.
